# Beyond the puff: qualitative insights into smoking behaviours and societal perceptions among university students in India

**DOI:** 10.1136/bmjopen-2025-101172

**Published:** 2025-06-24

**Authors:** Seemadevi Thangeswaran, Naganandini Sampath, Kumar Gaurav Chhabra, Pankaj Chaudhary, Avishek Singh, Shweta Dangi

**Affiliations:** 1 Public Health Dentistry, Nims Dental College & Hospital, NIMS University, Jaipur, Rajasthan, India; 2 Research & Development Cell, Datta Meghe Institute of Higher Education & Research, Wardha, Maharashtra, India

**Keywords:** Public health, Tobacco Use, Smoking Reduction, QUALITATIVE RESEARCH

## Abstract

**Abstract:**

**Objectives:**

The objective of the study was to understand the smoking behaviour of adults and how societal perceptions influence the smoking behaviour of university students.

**Design:**

Qualitative study.

**Setting:**

National Institute of Medical Sciences university, India.

**Participants:**

20 face-to-face interviews were carried out among university students who were in the age group of 19–30 years using a combination of purposive sampling, followed by snowball sampling methods.

**Results:**

Qualitative responses revealed that stress, cravings for cigarettes and mealtimes were key triggers for smoking behaviour. Many participants felt guilty about their smoking and often became irritated by advice from non-smoking friends. All participants had experienced negative health effects, including physical and sensory issues, as well as other adverse experiences. Students expressed a dislike for judgemental attitudes from society. They respected elders and found it difficult to smoke in front of them. Rather than being blamed for their smoking, they preferred supportive assistance to help them quit.

**Conclusions:**

The study highlights the importance of understanding college students’ smoking behaviour, as it greatly influences their smoking habits. Cessation efforts should target this group and emphasise the negative experiences associated with smoking. Additionally, students recommend creating a non-judgemental and supportive environment to aid in quitting, rather than a judgemental and blaming society.

**STRENGTHS AND LIMITATIONS OF THIS STUDY**

- A key strength of the study is its use of in-depth, face-to-face interviews, which provided rich, nuanced insights into students’ personal smoking experiences and societal influences.
- Significant advantage of this study lies in its qualitative approach, providing comprehensive insights into the factors and societal perceptions that shape the smoking behaviour of college students.
- A further strength is the inclusion of diverse participant perspectives, which enhances the credibility of the findings.
- One limitation of this study is the predominance of male participants, with limited involvement of female participants, which may affect the diversity and generalisability of the findings.
- One drawback of this study is that focus group discussions were not conducted, which could have provided valuable insights into group dynamics and collective perceptions regarding smoking behaviour.

## Introduction

 Tobacco is unique among consumer products in that, when used according to the manufacturers’ recommendations, it eventually causes the death of half its regular users.^1^ Smoking is widely recognised as the most significant preventable cause of death in numerous countries. According to the WHO, tobacco smoking is responsible for 6 million deaths each year.^2^ This alarming figure is expected to rise, reaching over 8 million deaths annually by 2030, if effective measures are not implemented to combat the issue.^2^ Young adults are the most valuable target for tobacco companies because, due to the addictive nature of tobacco products, attracting them to start smoking ensures many years of continued consumption, making it an exceptionally effective long-term investment. Tobacco companies should be held accountable for the loss of lives attributed to tobacco-related deaths. This is why they target young adults, aiming to establish a new generation of tobacco users, which perpetuates their profits despite the public health risks. India is the leading producer of tobacco, and a significant portion of its young adult population is addicted to this habit, largely due to tobacco companies targeting adult college students.^3^ Research shows that the earlier a person begins smoking, the more severe the health consequences they will face and the more challenging it will be for them to quit.^4 5^ According to the Global Adult Tobacco Survey 2016–2017, 42.4% of men, 14.2% of women and 28.6% of all adults currently either smoke tobacco or use smokeless tobacco. The average age at which individuals begin smoking falls within the 20–34 age range, with a mean initiation age of 18.9 years.^6^

Understanding the smoking behaviour of this adult population is crucial, as it significantly influences smoking habits in adults. While smoking behaviour can change dynamically throughout a person’s life, our knowledge of the motivations behind transitions in and out of smoking is limited, particularly regarding how these changes are linked to social relationships.^7^

One important aspect is that adult smoking behaviour is influenced by societal perceptions, which can have both positive and negative impacts. Several cross-sectional studies have indicated that societal stigma has led smokers to disapprove of their habit, which has contributed to successful quitting.^8^

It is important to understand that individuals have unique reasons for their smoking behaviour, and effective interventions should consider these factors to increase the likelihood of successful smoking cessation. According to Strauss and Corbin, qualitative methods are the most appropriate for uncovering the nature of people’s experiences and delving into the underlying factors behind them.^9 10^ Qualitative methods provide a pathway to begin uncovering adult smoking behaviour, offering the opportunity to gather comprehensive insights into their perspectives and the significance they attribute to smoking.^11 12^ This discovery will foster a thorough comprehension of how adolescents perceive smoking within the framework of their lives, which is essential for crafting effective antismoking interventions.

There is scarcity in research on how smoking behaviour in adults is shaped after the initiation of the habit and how societal perceptions impact their smoking patterns. The purpose of this study is to understand adult smoking behaviour and the influence of societal perceptions on it.

## Methods

The recruitment of 20 participants involved a combination of purposive and snowball sampling methods. Participants were selected from the Outpatient Department (OPD) of Oral Medicine and Radiology, NIMS (National Institute of Medical Sciences) University, Jaipur. Our study focused on college students who were smokers, aged over 18, Hindi and English speaking, and who provided consent to participate. To identify eligible participants, their smoking history was discussed, and after an oral cavity examination, they were chosen. The oral cavity of participants was examined for any lesions potentially linked to smoking, and their smoking history was confirmed. All participants were initially visiting the dental hospital for routine check-ups. Those with identified lesions were further counselled by the investigators 2, 3. Interviews were conducted during the dental institution’s OPD hours, between 9:00 and 16:00 hours, with participants recruited during both the morning and afternoon sessions. The study’s objectives were explained in detail, and participants were then taken to the college’s tobacco cessation centre. Demographic information about the participants was collected, excluding their names to ensure data confidentiality and anonymity. College students who had friends who were also smokers were encouraged to bring their friends into the study, achieving snowball sampling. Initially, a focus group discussion was planned, but it was discontinued due to concerns about social desirability effects and participants’ hesitance to express their answers. Instead, the approach shifted to conducting individual interviews.

The face-to-face interviews included questions about participants’ age, gender, year of study, professional course (stated by participants during demographic data collection) and their level of tobacco dependence, assessed using the Fagerström test for Nicotine Dependence level scale for smoking (online supplemental appendix 1). These interviews were semistructured and began with smoking behaviour based on the CAGE (Cut down or control, Annoyed or angry, Guilty, Eye-opener) behaviour scale (online supplemental appendix 2), followed by questions regarding how society sees them as a smoker. Additionally, participants shared about their smoking experience.

The interviews typically lasted for 10–15 min. Data collection is halted or discontinued when theoretical saturation has been achieved. This was assessed by the authors (investigators 1, 2, 3) when no new information was generated from the analyses.

The interviews were recorded using an MP3 recorder, while field notes were also taken during the interviews to capture additional observations and details that may not have been captured in the recordings. The ensuing data underwent transcription and analysis through a hybrid approach, combining inductive and deductive methods. Students’ smoking behaviour was assessed using the CAGE behaviour scale, which follows a deductive approach in qualitative analysis, and the examination of societal perceptions (as there is no validated scale to explore societal perceptions) was approached inductively, (as the information provided by the participants) allowing for the emergence of themes based on participants’ responses. Thematic framework analysis was conducted by two investigators. Conclusions drawn from the data were discussed among all three members of the research team (investigator 1, 2, 3, 4, 5, 6). All 20 interviews with notes were included as online supplemental appendix 2.

### Patient and public involvement

Patients or the public were not involved in the design, or conduct, or reporting, or dissemination plans of our research.

## Results

A total of 20 participants were interviewed for the study, 15 (75%) male and 5 (26%) female. The mean age of the participants was 24 years. The age range within the sample varied, with the youngest participant being 19 years old and the oldest being 30 years old. Distribution of participants based on the professional course mentioned in table 1.

**Table 1:** Distribution of students based on their professional course

Professional course	Male n (%)	Female n (%)
Engineering	3 (20)	2 (40)
Pharmacy	4 (26.6)	1 (20)
Dental	7 (46.6)	2 (40)
Law	1 (7)	0

Among the male participants, 53% were classified as minimally dependent. Among the female participants, 60% were classified as moderately dependent. Notably, none of the participants, regardless of their gender, were categorised as highly dependent. It highlights the prevalence of minimally and moderately dependent individuals within the sample. The dependence level of the participants is described in table 2.

**Table 2:** Description of Nicotine dependence level based on gender of the students

	Dependence level			
Sex	Minimally dependent n (%)	Moderately dependent n (%)	Highly dependent n (%)	Not dependent n (%)
Male (15)	8 (53.3)	5 (33.3)	0	2 (13.3)
Female (5)	2 (40)	3 (60)	0	0

### Smoking behaviour

#### Stress and emotional triggers

Some participants mentioned that stress, anxiety and frustration trigger their cravings for cigarettes. This suggests that smoking serves as a coping mechanism for dealing with difficult emotions.

Yes, sometimes when I'm stressed out, I have cravings for cigarettes”. I find it challenging at times. It could be that when I'm stressed or anxious, I become irritable at work, and if I have a strong craving for a cigarette at that moment, it can create problems. However, this doesn't happen very often; it’s rather unusual (30s female/moderately dependent)

I only smoke when I'm feeling sad…When I'm at home, I typically smoke only one cigarette at night. However, if I don't have one, it can make me anxious. This is especially the case because I tend to overthink and have issues with anxiety (20s female/minimally dependent)

### Independence and defensiveness

Participants expressed a strong sense of autonomy regarding their smoking, viewing it as a personal choice that should not be subject to external judgement. They often displayed defensiveness towards criticism, particularly from others who disapproved of their smoking habits. Some participants expressed frustration with societal judgements, highlighting their belief that their personal choices should not concern others. This defensiveness reflected a desire for independence, as participants rejected interference from outside opinions.

Yes, I see it as my personal choice, and I don't appreciate others interfering in my decisions. I would advise them to mind their own business (20s female /minimally dependent)

I smoke with my money and I'm not bothered by others' opinions or judgments (20s male/minimally dependent)

Many people criticize my smoking and say it’s bad, which is frustrating for me…I'm not using their money to purchase cigarettes, so it shouldn't concern them (20s male/minimally dependent)

#### Irritation at advice

Participants expressed irritation when advised to quit smoking, highlighting resistance to external influence. They are more receptive to well-informed advice but reject criticism.

don’t ask me about it… all the times, I have friends who haven’t even touched a cigarette in their entire life. they know I’m having this bad habit but somewhere or the other they come to ask me…I know it is for my benefit that they are saying but sometimes it is irritating……. You do your business; I will do my own… you don’t have to come and counsel me (20s female/minimally dependent)

I stay with people who smoke, and I don't appreciate it when others advise me to quit (20s male/moderately dependent)

Yes ……specially girls when they tell not to smoke, I feel irritated (20s male/minimally dependent)

I don't get angry with my friends for their concerns, but in the past, when I was staying in a hostel, an aunty there criticized me and imposed a strict no-smoking rule. It became uncomfortable for me, so I chose to leave that hostel because I couldn't smoke there (20s female/minimally dependent)

### Guilty about smoking

Participants frequently reported feelings of guilt associated with their smoking habits, coupled with a strong desire to control or quit. Despite this intention, many acknowledged the challenge of quitting and admitted to returning to smoking after attempts to stop. This tension between guilt and the struggle for control highlights the complexity of overcoming smoking addiction.

I experience guilt because smoking is not a good habit, and it also comes at a financial cost. I often think I could have invested that money in a more worthwhile way, and that’s why I feel guilty at times (20s female/minimally dependent)

More than my guilt about the past, I'm concerned about the future. I worry about not being able to quit this habit, as I had planned to quit once I return home and finish my college. What if I can't quit, and someone at home discovers me? I'm aware that my sibling also smokes, but everyone has their own way of hiding it. This future uncertainty is a significant source of concern for me (20s female/minimally dependent)

### Smoking patterns association with meal

Many participants reported a strong association between smoking and mealtimes, particularly after breakfast, lunch and dinner. For some, smoking was a habitual part of their routine, with one participant noting the need for a cigarette after every meal. Morning smoking was also common, with several participants mentioning that they typically smoked after waking up. These patterns suggest that mealtimes and the start of the day serve as key triggers for smoking, reinforcing the challenge of quitting as smoking becomes intertwined with daily routines.

After every meal, including breakfast, lunch, and dinner, I feel the need for a cigarette (20s male/minimally dependent)

I don't consider myself a morning smoker. I have friends who wake up and immediately think about cigarettes, but I'm not at that point. I get ready and start my day without craving a cigarette. However, as the day progresses, certain triggers can make me want to smoke. It could be anything like conflicts with colleagues or seniors, receiving instructions, good or bad days, or dealing with stressful situations like staff members scolding me. While I may need 1 or 2 cigarettes during college hours, the desire to smoke increases when there’s a specific trigger or a challenging situation (20s female/minimally dependent)

In the morning, I must have one cigarette, and as the day progresses, I have them with tea and other meals (20s male / moderately dependent)

If I have breakfast, I tend to smoke, but if I skip breakfast, I don't (20s male/minimally dependent)

#### Availability of cigarettes

Participants emphasised that the availability of cigarettes significantly influenced their smoking behaviour. For many, smoking was directly tied to whether they had access to cigarettes; if they had them, they smoked regularly, and if they didn’t, they refrained. Instances where access was restricted, such as during the COVID-19 lockdown, provided opportunities for participants to control their smoking habits. Additionally, social context played a role, as participants reported smoking more when outside of college, suggesting that the company and environment influenced their smoking patterns.

My smoking pattern is solely based on availability. If I have cigarettes for 24 hours, I'll smoke for 24 hours. If I don't have them, I won't smoke, as simple as that (20s male/moderately dependent)

During the COVID times, I didn't have access to cigarettes, so I tried to control my habit (20 s male/minimally dependent)

It depends on the company I'm with…… In college, I often don't have access to cigarettes, but if I'm outside of college, I tend to smoke (20s male/minimally dependent)

#### Family influence

Participants highlighted the significant influence of family on their smoking behaviour. Many avoided smoking at home or in the presence of family members, particularly older individuals, out of respect or to prevent judgement. The presence of family members, such as parents, also acted as a trigger for feelings of guilt, motivating some participants to consider quitting. This suggests that family dynamics and interactions play an essential role in shaping attitudes and behaviours around smoking.

I don't smoke at home; I only smoke here at my college. When there are older individuals or family members present, I refrain from smoking and stay away from it (20s male/moderately dependent)

Whenever daddy sits next to me smell will be coming that time, I want to quit ……… (20s male/moderately dependent)

#### Future intentions

Participants expressed mixed future intentions regarding smoking. Many showed a desire to quit, often setting resolutions such as at the start of a new year, but struggled to follow through due to various challenges. Others reflected on the harmful effects of smoking and emphasised the importance of discouraging future generations from adopting the habit. These intentions highlight both personal struggles with quitting and a broader awareness of the need to prevent smoking in younger populations.

I often feel like quitting every New Year, but I struggle to stick to that resolution (20s male/minimally dependent)

I firmly believe that the upcoming generation should refrain from smoking as it is harmful to health (20s male/ moderately dependent)

A wide range of themes expressed in relation to smoking behaviour of students. This in-depth analysis reveals the multifaceted nature of smoking behaviour, with stress relief, social influence, guilt and personal autonomy playing significant roles. Understanding these complexities can inform strategies to support those who wish to quit smoking and provide insights for health interventions. It also underscores the need for a holistic approach that takes into account the emotional, social and personal aspects of smoking habits. These themes reflect the diverse attitudes, experiences and challenges associated with smoking behaviour among the participants. This brief analysis highlights the emotional, social, and personal aspects of smoking behaviour and offers insight into the complexities involved in the participants’ experiences. Understanding these factors is important for designing effective smoking cessation interventions and support.

#### Experience and feelings and effects related to the individual’s smoking habit

Many people reported experiencing adverse physical effects from smoking, including a bad taste in their mouths, unusual tastes when eating, coughing, breathing difficulties and dizziness while doing exercises, sore throat, burning sensation. The participant shares an alarming experience of fainting during a smoking session in cold weather, indicating the potential health risks and dangers associated with smoking. Table 3 summarises themes based on students’ experience on smoking habits.

**Table 3:** Description of themes on smoking experience

Themes	Responses from participants
Physical and sensory effects	“After smoking 2–3 cigarettes a day, I notice that my mouth develops an unpleasant taste, and when I eat, the flavors of the food seem weird”.“(20 s male/minimally dependent)“I initially started smoking out of curiosity to give it a try. I find it relaxing when I smoke. On one occasion, I experienced the presence of black stains in my sputum” (20s male/moderately dependent)“During exercise or running, I struggle with breathing, and at times, I experience excessive coughing and the expulsion of sputum. I also face breathing difficulties when climbing up and down stairs” (20 s male/moderately dependent)“For me, smoking is enjoyable when paired with drinks, but it comes with the downside of a sore throat and a burning sensation. I also tend to feel dizzy while smoking (20s male/ moderately dependent)
Adverse experience	“One bad experience is there, Once I was at my friend’s place and it was winter time…we were smoking and I was wearing a puffer jacket and I started sweating so I went outside, in the balcony…then I fell unconscious…” (20 s female / moderately dependent)
Social influence /peer pressure	“Smoking felt enjoyable to me, but when someone judged me for it, it made me feel uncomfortable. Initially, I began by taking cigarettes from my friends. However, as time went on, my friends encouraged me to buy my own and have the experience. It’s worth noting that, in my experience, it was somewhat of a societal taboo for girls to go to the shop to buy cigarettes” (27 s female/minimally dependent)
Influence of media/coping of stress	“Smoking brought us enjoyment, particularly when we were listening to our favorite songs. This often led to smoking more, and sometimes, I would even copy certain actors. …. it made me feel happy” (20 s old male/minimally dependent)
Changing environments /social norms	“Places where smoking is restricted…people tell not to smoke and earlier there was not much strictness in my university campus and I could smoke wherever I felt like but now there is too much restriction” (20s male minimally dependent)
Initial experiences and progression	“When I first began, I only had one cigarette, and then I stopped for a while. However, for the past year, I have resumed smoking. During this time, I typically smoke with my friends, and I find it to be good experience” (20s male /minimally dependent)

The participant also describes how the influence of friends played a significant role in their smoking habit. Friends initially provided cigarettes, and peer pressure to buy their own cigarettes eventually influenced the participant.

Some participants mention that they feel happy and relaxed when smoking, particularly when listening to their favourite songs by copying actors. Smoking is used as a coping mechanism to reduce stress and tension for participants.

Some individuals reported that changes in smoking habits are influenced by environmental factors, such as restrictions in certain places. Stricter rules in university campuses have affected where and when individuals can smoke.

Some participants enjoyed it in the beginning, but recent experiences have been less enjoyable. One individual describes their smoking journey, starting with one cigarette, leaving it for a while, and then returning to smoking.

Thus, illustrate the complex relationship between smoking and various factors, including social influences, stress relief, sensory experiences and evolving attitudes towards smoking in different environments. The participant’s awareness of health risks and mixed feelings about smoking is also evident.

### Societal perceptions related to smoking habits of students

#### Societal judgement

Several individuals mentioned that they feel judged by society for their smoking habits. Society often views them as ‘cringe’ or ‘rogue’ individuals who lack manners or better things to do.

In society’s eyes, I might be perceived as a cringe person or a rebel. Some may think of me as someone lacking manners or having nothing better to do (20s male/minimally dependent)

#### Indifference to others’ opinions

Some individuals express a lack of concern for what others think about their smoking habits.They emphasise that they do not care about societal judgement and prioritise their personal choices.

I've reached a point in my life where I don’t really concern myself with what other people think about me. Their opinions don't matter to me (20s male/minimally dependent)

#### Respect for elders and role models

A few individuals mentioned that they hide their smoking in front of elderly people out of respect. They consider it a matter of principles and cultural values.

it’s not just about smoking; I generally don't think much about what people think of me. However, when I come across an elderly person who is around the age of my parents while I'm smoking, I tend to hide my cigarette. This is something I’ve learned from my elders out of respect. I apply this behavior whether I know the person or not, and I also don't smoke in front of my parents (20s female/minimally dependent)

#### Professional and social consequences

Some individuals acknowledged that their smoking is seen negatively, especially in their professional or social circles. Smoking is viewed as a behaviour that sets a bad example for society, and they may face criticism.

initially it mattered to me…like obviously being a doctor and smoking people won’t think good about me but it doesn’t matter what they are thinking…I know being a doctor I should not(20s male / minimally dependent)

it matters we are in good profession its bad for me and for the society too we are setting a bad example for society(20s male/ minimally dependent)

#### Gender and smoking

The gender aspect is highlighted in some statements. People may have different reactions to women who smoke, such as feeling surprised or seeing them as less conventional.

ok it is wrong but you don’t have to make the person smoking feel bad…walk from a distance, they’ll make faces as if I am going to buy a cigarette and there are guys around they’ll give me looks like oh she is a girl and buying cigarettes all these things annoy me …you know (20s female /moderately dependent)

#### Age and peer perception

Age plays a role in how individuals are perceived when smoking. Younger peers might see it as ‘cool’, while older individuals, especially elders, may view it negatively.

people my age think of me as a student and cool when they see me smoking but older people don’t see me nicely (20 s male minimally dependent)

#### Social pressure to quit

Some individuals mentioned that societal pressure or disapproval is a motivating factor to quit smoking. They feel constrained by societal norms and expectations.

if the society is smoker they see me very nicely and if they are nonsmoker then they’ll see me with bad and cruel eyes. It is specially annoying when I am smoking and someone crossing me will cover their mouth (20s female /moderately dependent).

I smoke in university because nobody cares; most people here are around my age and smoke as well. However, I don't smoke at home because people there would look at me as if I'm committing a crime (20s male/minimally dependent)

Some individuals mentioned that societal pressure or disapproval is a motivating factor to quit smoking. They feel constrained by societal norms and expectations. These themes demonstrate the complex interplay between personal choices, societal expectations and cultural norms in relation to smoking. It reflects the diverse attitudes and experiences of individuals who smoke within the broader context of society.

## Discussion

Several cross-sectional studies aimed to evaluate the smoking behaviour of students.^13 14^ A qualitative study conducted by Nichter *et al* explored smoking behaviour in adults and identified stress as a significant factor contributing to smoking habits. These findings are consistent with the results of our study, which similarly recognised stress as a key driver of smoking behaviour among university students.^15^ Similarly, Al-Natour Ahlem *et al* conducted a qualitative study focusing on the perceptions of smoking among female Jordanian university students. Their study highlighted peer pressure and stress as key factors intensifying smoking behaviour, which mirrors the experiences shared by participants in our study.^16^ These parallels underscore the universality of stress and social influences as driving forces behind smoking, irrespective of demographic or cultural contexts. A qualitative study investigating the barriers to smoking cessation among university students highlighted peer influence and stress as major obstacles.^17^ Participants in that study frequently reported that social pressure from peers and the overwhelming burden of stress made it particularly challenging to quit smoking. Similarly, our study found that stress acts as a critical trigger for smoking behaviour, with many participants indicating that they resort to smoking as a strategy to manage or cope with their stress. These findings underscore the intertwined roles of stress and social dynamics in perpetuating smoking habits among university students, emphasising the need for targeted interventions that address these underlying factors. Furthermore, some participants expressed irritability when receiving advice from non-smoker friends and defensiveness in response to criticism of their smoking. They also revealed feelings of guilt about their smoking behaviour and a reluctance to expose their habit to their families, often attempting to hide it from them. Therefore, it may be more effective for cessation counselling to adopt a family-centric approach rather than providing advice and counselling, as many individuals seem irritated by such advice, especially when targeting adult populations.

Our study also found a connection between smoking and meal associations among smoking college students. Among the moderately dependent smokers in our study, it was revealed that they tend to smoke in the morning and have a strong association between smoking and meals, particularly with breakfast. Additionally, they emphasised that the availability of cigarettes plays a significant role in their smoking habits. During times of the COVID-19 pandemic, some participants faced difficulty in accessing cigarettes, which led them to make efforts to control their smoking habit. These findings underscore the importance of cessation services carefully evaluating participants’ behaviours and selecting the appropriate cessation methods. A personalised approach tailored to individual needs and habits is crucial in helping individuals successfully quit smoking.

Our study findings explored the perspective of students who smoke regarding how society perceives them. They expressed their discomfort with the judgement passed on students who smoke and revealed that instead of criticism, they need advice and support from society. Many of these students hold a deep respect for their elders and are hesitant to accept criticism from the broader society. To the best of our knowledge, this study is the first to document the viewpoints of students regarding society’s view of smoking. This social aspect is highly significant and can be positively integrated, such as through public education initiatives about smoking, in which the public can actively participate in smoking cessation efforts.

One of the strengths of this study is its qualitative approach, which provides rich, in-depth insights into the smoking behaviour of college students. By focusing on this demographic, the study addresses a critical age group that is at high risk of initiating smoking and developing long-term habits. The study also explores various factors influencing smoking, such as stress, societal perceptions and smoking triggers, which contribute to a holistic understanding of student behaviour. Additionally, the study offers practical relevance, as its findings can directly inform interventions and policies aimed at helping college students quit smoking, a group that is often underrepresented in smoking cessation research.

A key limitation of the study is its small sample size of 20 participants, which may limit the generalisability of the findings to a larger student population. The gender imbalance in the sample, with a higher number of male participants, further restricts the ability to fully understand female students’ smoking behaviours, as fewer females participated in the study. Additionally, conducting the study at a single institution and limited disciplines involvement may not fully capture the diversity of smoking behaviours seen across different regions or types of academic institutions. Another limitation is the reliance on self-reported data, which can introduce bias, as participants might underreport or misrepresent their smoking habits due to social desirability issues.

The findings of this study have important implications for public health policies targeting smoking cessation among college students. Cessation programmes should be tailored to address the unique triggers that affect students, such as stress, social influences and peer behaviours. Additionally, interventions should focus on fostering a supportive and non-judgemental environment where students feel comfortable seeking help to quit, rather than creating a climate of blame and criticism. Universities should consider incorporating smoking cessation resources into their wellness programmes, making them easily accessible to students. Furthermore, policies restricting tobacco availability (Tobacco Free Educational Institutions, TOFEI) around college campuses could help limit students’ access to cigarettes, thus reducing the temptation to smoke, especially during stressful periods.

Future research should focus on expanding the sample size and ensuring greater diversity in terms of gender, ethnicity, and academic disciplines to improve generalisability. Longitudinal studies are needed to track smoking behaviours over time and assess the long-term impact of cessation interventions. Research across multiple institutions would provide valuable insights into regional and institutional differences. Additionally, studies should explore gender-specific factors influencing smoking behaviour and test non-judgemental, supportive interventions in college settings. Finally, research evaluating the role of social and environmental factors, such as peer pressure and stress, in smoking habits could inform more effective prevention and cessation strategies.

We, as investigators, were vigilant during data collection and interpretation, ensuring a neutral and unbiased approach. Building rapport with participants was essential to make them feel comfortable discussing sensitive topics such as smoking. We remained conscious of potential biases and took proactive steps to minimise their influence, ensuring the findings accurately represented the participants’ perspectives. Regular reflection throughout the process helped maintain objectivity and integrity in the study.

In this qualitative research study, college students shared their perspectives on smoking. The findings from this research hold significance for educators and healthcare professionals, particularly those dedicated to antismoking initiatives and health promotion. They can use these findings to aid in the creation of intervention programmes that are tailored to the needs of smoking college students, ultimately enhancing their effectiveness in addressing the issue of smoking

## Conclusions

This study highlights the critical need to understand college students’ smoking behaviour, as it plays a significant role in shaping long-term habits. Cessation efforts should prioritise this group, emphasising the negative health effects of smoking. Students also recommend fostering a non-judgemental and supportive environment to aid in quitting, rather than a society that blames and criticises smokers. Public health policies should incorporate stress management strategies and create supportive spaces within academic institutions to help students quit smoking. By addressing these factors, we can work towards reducing tobacco use and preventing long-term addiction among young adults

### Public health significance

Tobacco cessation services are often designed for the general population, lacking age-specific approaches, which can make them less effective, especially for students. Therefore, it is essential to develop tailored cessation services specifically for the student population. Students have unique perspectives on smoking, so engaging with them to understand their smoking behaviours and experiences can help create customised services that are more effective in promoting cessation. ToFEI should be implemented in all universities.

## Supplementary material

10.1136/bmjopen-2025-101172 online supplemental file 1

10.1136/bmjopen-2025-101172 online supplemental file 2

## Data Availability

All data relevant to the study are included in the article or uploaded as supplementary information.
