# Supplementary material for: Beyond the puff: qualitative insights into smoking behaviours and societal perceptions among university students in India
Source: BMJ Open. 2025 Jun 24;15(6):e101172. doi: 10.1136/bmjopen-2025-101172 (PMC12198830; doi:10.1136/bmjopen-2025-101172)
Supplement: online supplemental file 1 [file bmjopen-15-6-s001.docx]

Nicotine Dependence level was assessed using **Fagerstorm Test for Nicotine Dependence [FTND] scale**

1. How soon after you wake up do you smoke your first cigarette?

3 - Within 5 minutes

2 - 6-30 minutes

1 - 31-60 minutes

0 - After 60 minutes

1. Do you find it difficult to refrain from smoking in places where it is forbidden (e.g. in church, at the library, cinema, etc.)?

1 - Yes

0 – No

1. Which cigarette would you hate to give up?

1 - The first one in the morning

0 - All the others

1. How many cigarettes/day do you smoke?

0 - 10 or less

1 - 11-20

2 - 21-30

3 - 31 or more

1. Do you smoke more frequently during the first hours after waking than during the rest of the day?

1 – Yes

0 - No

1. Do you smoke if you are so ill you are in bed most of the day?

1 – Yes & 0 – No

**CAGE Questionnaire Modified for Smoking Behaviour***

| 1. Have you ever felt a need to **C**ut down or control your smoking, but had difficulty doing so? |
| --- |
| 2. Do you ever get **A**nnoyed or angry with people who criticize your smoking or tell you that you ought to quit smoking? |
| 3. Have you ever felt **G**uilty about your smoking or about something you did while smoking? |
| 4. Do you ever smoke within half an hour of waking up (**E**ye-opener)? |
