# Supplementary material for: Beyond the puff: qualitative insights into smoking behaviours and societal perceptions among university students in India
Source: BMJ Open. 2025 Jun 24;15(6):e101172. doi: 10.1136/bmjopen-2025-101172 (PMC12198830; doi:10.1136/bmjopen-2025-101172)
Supplement: online supplemental file 2 [file bmjopen-15-6-s002.docx]

**Qualitative study interviews**

**I:** when did u first started smoking

a: 3 months ago

I : before that did u tried

a : last 5 years only 4-5 puffs

I : now why u started

a: in NIMS nothing else is there to timepass and all the friends are also smoking so they forcibly made me to start

I : 4-5 stuffs also with friends

a: yes

i: is there any difference do u feel when u started before and now

**a :** yes it feels different before it was fun and now its normal

i : if u feel normal why u still smoke

a: there no other option we were sitting outside at one place what to do

i: as such u don’t feel good or u feel high when you smoke

a : I feels high but regret is more

i: how you get cigarettes and how you buy cigarettes like complete packet or loose cigarettes

a : 1 cigarettes

i: do you know that loose cigarettes are banned

a: hmm

i: have you felt urge to control the smoking

a: daily

I : why u r not able to control

A: there is no other work we are sitting outside everyone’s smoking there and then friends give cigarettes in hand then I take it

I : have u ever get annoyed when any of your friend told you u to quit

A: no I doesn’t get annoyed

I: what u feel if someone interrupts u and if someone throws your complete cigarette

A: I don’t feel angry It feels good because somehow, I also want to control but I’m not able its automatically happening

I: how u ever went to guilt trap about coming to this college and having this friend circle because of which u stated smoking

A: I don’t have any guilt trip about friend’s circle but I have guilt trip that I stated smoking

I: anything u want to add regarding guilt trip

A: those people who are not smoking can help us to reduce our cigarettes

I: after waking up when is the first urge to smoke

A: no not in the morning I feel in the afternoon to smoke

I: do u smoke during the college hours also

A: yes

I: is there any association between any of your meal

A: no nothing like that it’s just when I’m free I smoke , when I’m busy I don’t smoke

I : what reason do u feel that u want to quit the habit

A: main reason is smelling is there so other person also feels uncomfortable

I: which is stopping u to quit

A: I have more free time there is no 2^nd^ option

I: how society sees u when u smoke

P : im at this point of life where what other people think about me I don’t think about that opinion doesn’t matters to me

I: do u want to quit the smoking

A: hann

I: why

A: I know the drawbacks

I: what drawbacks u know

A: cancer, it come at later stages, main thing is bad breadth

I: do u know about tobacco cessation center , what u know

A: I have heard the name but I don’t know what are therapies in there

I : any suggestions to improve TCC?

A: ppl considered smoking as a cool statement because of this ppl not utilizing tobacco cessation center

**INTERVIEW**

I: When u started smoking at what age

B : 21

I : HOW

B : just by seeing

I: whom u saw

B: friends

I: what u had tried first

B: cigarettes

I: how you got access to cigarettes

B: from friends ,one of my friends was having I took it from him

I: how was the feeling is it good or bad

B: first time it was heavy , nashey ( dizziness) I cough I took only 1-2 puffs

I: experiences ?

B: I get irritated by cigarettes

I: why

B: after taking 2-3 cigarretes in a day my mouth tastes bad and when I take food it tastes weird

I:bad experenices

B: whenever I go home I don’t smoke and when I came back I do smoke its weird

I:how many cigarress in ad day now

B: I quit smoking

I: When did u quit

B: 3- 4 month back

I: how many cigarettes did u smoke 3-4 months back

B: 2-3 cigarrees in a day after dinner when I get tired

I: did u buy the entire packet of cigarretes

B: my friends buy I’ll take 1-2 from them

I: control

B:its not like that ill smoke when I feel like smoking otherwise I won’t

I:angry

B: no I never felt angry

I: guilt

B: whenever its Sunday ,I smoke with friends and if I smoke more than 3 then I feel bad

I:wakeup

B: ill smoke when I feel like smoking, evening time its more with tea or alcohol

I:which factor motivates u to quit

B: when I went home that time I felt itching (kaarash) in my throat so I decided to quit and I felt good

When I smoking that time its feels good after that I felt tired and when I quit it I feel good

I: society

B: ill smoke in university nobody cares because everyone here around my age idont smoke in home ppl will look at me as if im committing crime

I: tobacco cessation center

B: yes , for quitting smoking ,nicotine

I: tcc therapy

B:no

I:opinions

B: till someone doesn’t go themselves then its no use if someone told to quit also they wont quit until they wont feel confident about themselves, theres no motivation I got irritated by cigarrees then I quit it

I: suggestions

B: teachers and doctors sitting in tobacco cessation center they take it lightly and they wont see the pt as they will quit so they wont motivate good

I: …

B:I have smoked before , and when I quit I felt good so no one should not smoke

**INTERVIEW**

**I:** when u started smoking

P: as far I remember I was in introduced to smoking after 12^th^ class normally when I used to go for tuitions

I: who

P: peers only people who used to tuition along with me they used to smoke and taking my own cigarette is started

I; how many

P: it can go to 5 I don’t smoke very much in weekends it can go to 7,8i don’t cross 10

I: duration

P: I passed my 12 th class in 2012,now its 2023

I: how was the experience

P:it is good if somebody judges me I feel bad ,first I started my cigarettes it was bet I take from my friends then friends started telling me go purchase your own and experience it usually girls never used to go shop it’s a taboo

I: access

P:,if its city I will go and get it if its small cities I will get from guy friends

I:control

P: many times , I feel like quiting it when it is in my hands its very difficult , I will be determined I will quit from tomorrow and if see my friends then it becomes difficult . its not like I don’t feel like quitting it . its just I am not able to take myself there

I: anger

P:don’t ask me about it.. all the times ,I have friends who haven’t even touched a cigarette in their entire life. they know im having this bad habit but somewhere or the other they come to ask me…I know it is for my benefit that they are saying but sometimes it is irritating.you do your business,I will do my own . you don’t have to come and counsel me

I; guilt

P; more than past I have guilt of the future...what if I am not able to ultimately stop this habit…because I think I will quit when I reach home and finish my college but what if I am not able to quit and somebody at home catches me…I know my sibling also do it but everyone has their way of hiding it.

I; time

P; no I don’t think I am a morning smoker. I have my friends who wake up and think of cigrettes..i have not reached there yet. I get ready and start my day then ill have some trigger which will make me want to smoke. Anything like fight will colleges or seniors giving instructions …somedays are good or bad …staffs shouting at you.some trouble will come and then ill want to relax…I wont say it is only in college hours…I need 1 or 2 in college hours but yes if there is a factor it will go more

I don’t want it in the morning but when the day kick starts ill need it

I; motivation

P; as I told.. thinking of what if in future otherwise I am normal.i am afraid of the fact that I ll become an addict.. What if I get married and my partner is against it then thinking of that…what if my children get this habit from me…thinking of any such reasons drives me crazy

I; society

P; if the society is smoker they see me very nicely and if they are nonsmoker then they’ll see me with bad and cruel eyes. It is specially annoying when I am smoking and someone crossing me will cover their mouth. ok it is wrong but you don’t have to make the person smoking feel bad…walk from a distance, they’ll make faces as if I am going to buy a cigarette and there are guys around they’ll give me looks like oh she is a girl and buying cigarettes all these things annoy me …you know

I; want to quit

P; yes …no I don’t think I want to quit it..i guess I need to

I; tcc (tobacco cessation center)

P; yes I have one in my college..thats how I know about it..people sit there and counsel…I myself did it being a doctor…

I; tcc therepies

P; I myself have consouled people so I know those therapies but I don’t think they work

I; any suggestions

P; just because they have not reached there yet..they don’t realize also I myself don’t realize how grave this habit is..people don’t know they are in trouble and if they know they don’t want to get out of it..maybe this is the reason …I don’t find myself in the position

**Interview**

I: when u stated smoking

K: when I was small my grandfather used to smoke hookah ill stated that time only then on family gathering I had hookah then my grandfather used to have bidi whenever he goes out ill have bidi after that I came for studies when I was around 19 year since I have been smoking constantly

I: what reason

K: with friends

I: experiences

K; when listening to my favourite songs ill smoke more and copy some actors

I; what all have you smoked

K; bidi,ciggrettes,hookah,chillum,charas,gaanja

I; access

K; friends made me try and I enjoyed. some areas like railway station or bus stand,slum areas where they would sell it

I; difference and what was better

K; cigrettes made me have continuous loose motions I would visit doctor regularly. I had paan masala also it gave me ulcers in mouth,hookah made me cough

I; what are you smoking currently

K; cigrettes and on shivratri I take Ganja

I; how do you buy

K; single everyday at night after dinner of 10rs

I; you know buying a sinlge ciggrette is banned in india

K; no I had no idea

I; how many in a day

K; sometimes no at all if with friends then I have even taken an entire packet in one go

I; control

K; I feel like quitting from every new year I feel something in my throat

I; why cant quit

K; sometimes I feel sick in stomach and cannot be fresh in morning . constipated for days

I; angry

K; yes specially girls when they tell no to smoke I feel irritated

I; guilt

K; yes when I look at a healthy person then I think about why I started and I should leave

I; time when urge

K; at night between 12 to 1 after dinner or if in morning then after a heavy breakfast

I; with food or what

K; with tea 1-2 years back

I ; what motivation

K; if I compare myself with a healthy person who is fit because I am fat then I feel bad and I cannot go to gym as I become short of breath and I also feel my blood pressure fluctuating. My hands and face sweat a lot

I; why not able to quit

K; I feel like and then I get influenced by friends also…I also like drinking with them

I; perception of you

K; I stay with people who smoke and I don’t like people who interrupt me or tell me to quit

I; you want to leave

K; I want to quit only if I get better after it and my stomach issues resolve

I; tcc

K; no

I; tcc therapy

K; no I am not aware

I; any suggestions

K; I know my health is not good I feel a lot of sputum..3-4 days before I noticed dark sputum

I; improve tcc

K; there should be someone to talk to like in my case sometimes lonliness makes me do it or after food I feel it will not be digested if I do not smoke so there should be awareness about it

**Interview**

I: when u stated smoking

6:9years back

I:How

6:While hanging out with friends...One was smoker who introduced

I:What did you smoke

6:Cigarette

I:What all have you smoked

6:Hookah, cigarettes

I:Frequency

6:Now it has reduced now ....2-3 times in a day... morning and evening

I:Peak

6:5-6 cigarettes in a day

I:Experience

6:I felt happy and it was good ...we enjoyed it

I:Why reduced now?

6:It's not a good thing and it's consuming my health now

I:How is it affecting your health

6:As an athlete, I can't perform well... I get tired easily...I think smoking is the reason

I:Access

6:From a shop in nearby area

I:Packet or single

6:At First I had single then I got addicted and now i buy packets

I:You know that loose cigarettes are banned

6:No

I:Control

6:Yeah Many times

I:Problems you face

6:Company and sometimes when I am alone, I get bored and stat to smoke but mostly it is because of company

I:Angry

6:Some people want to smoke...and if someone stops, I take it in a good way.

I:Do they advise or criticize

6:they mostly say "stop it is not good for your health"Yes they advice and not criticize

i:Guilt

6:No...I don't think so

I:Timings

6:If I have breakfast then I smoke...if not then I don't

I:Association with meal

6:It depends on company usually in college theres no access...if I am outside, I smoke

I: with Tea

6:No mostly it depends on company....if I am with friends I smoke if I am alone I won't

I:Emotion

6:If I am sad then only

I:Do you want to quit it

6:Yeah

I:What motivates you

6:My health...I want good health

I:What is stopping you

6:Lack of motivation is there ....I am not getting motivated

I:Society

6:I don't smoke in public until and unless they are smokers...if it is an unknown place I will prefer not to smoke

I:Tcc

6:No

I:Tcc therapy

6:No I have only heard of nicotine chewing gums

I:Suggestions

6:Nobody came to me as of now...I think there should be programs and people should be made aware

**INTERVIEW**

I: when u started smoking

A: when I was in 12 th class…my one exam really bad and I was anxious and worried about the next one so my younger brother insisted me to smoke…he said I would feel calm and it will stop your overthinking as well. So I smoked and to my surprise the next exam went very well ….so that was the very first time.but after that I didn’t do it until I came to college. I started like regular smoking in my second year.

I; what did you smoke

A; cigarette only

I; what all have you smoked

A; cigrettes,marijuana

I; with whom do you smoke

A; friends in college…I was stressed initially so I did but then I got such a company . I do not blame anyone…it was my choice only

I; how many per day

A; with friends or if I am alone and bored then I smoke otherwise if I am busy then I don’t. when I smoke I take about 4-5 cigrettes per day

I; packet or single

A; no not a packet…3-4 cigrettes loose

I; how do get access

A; nearby shops

I; morning urge

A; if I have a task which requires a lot of concentration then I do in the morning otherwise not..like today I had a seminar so I had in the morning.

I; food association

A; after having food ..i usually do smoke. There is a craving after I eat something.

I; storage

A; yes I keep at my room only

I; control

A; no I have never willing thought that.. but during covid times, I had no access to it…so I thought of controlling but willing I have never thought of quitting

I; challenges

A; when at home,I smoke only one cigarette at night…but when I don’t get it…because I am an overthinker and I have anxiety issues …I feel anxious.

I; angry

A; not with friends…but earlier I used to stay in a pg and an aunty there was critising me and saying no cigrettes allowed. So I left that pg because I could not smoke there

I; guilt

A; I feel guilty as it is not a good habit also they come with a cost so I could’ve invested that money somewhere else. That is why I feel guilty sometimes

I; do you wake up and smoke

A; not everyday only if I have to study something or if I have exams

I; motivates

A; because everyday says it is bad and it costs me 40-50 rs everday. Obviously it is not a good habit but I also feel that after college we all have to live a monotonous life so…and I also feel that I womt be able to do it after college so I do…

I; what is stopping

A; i want quit it ..yes…but I haven’t thought of it willingly because quitting will be good or I want to reduce the amount as I know I wont be able to leave in one go..but I want to reduce it

I; society

A; initially it mattered to me…like obviously being a doctor and smoking people wont think good of me but it doesn’t matter what they are thinking…I know being a doctor I should not.

I; tcc

A; yes I know about it..it is in our college also…many times I have thought of going there and asking for help but somewhere I think if anyone will help me in quitting

I; tcc therapy or any friends had

A; no I haven’t heard any of them… some of my friends tried the nicotine patch but their experience was not good some felt like vomiting…they say once you are addicted to smoking then you get addicted to the patches so they don’t work. I have not tried patches and all

I; suggestions

A; is is very good and it should be the primary focus as many students and youngsters they are into smoking…and if you get into it,it becomes a helpless situation and if you want to get out you cant

I; what the doctors should do

A; doctors can explain the ill effects but I think I doesn’t matter to the smoker because on the cigarette packet also there is that photo of cancer but I don’t know maybe because of the composition or the nicotine in it…to the smokers they think that once I smoke then notjhing will happen but I think it is on the will of the person …if the person want to quit and is like fully convicted then tcc should take charge and help the person to quit

I; suggestions

A; also,in tcc… I thought 2-3 times…but my friends told me that they are very costly so there should be services that provide it for lower cost or free

**Interview**

I; start

R; when I was 21…when I was in 2^nd^ year..one of my friend told me to try…I felt very good..hallucination type…4-5 friends were there in the group who used to smoke and I was new to it.

I; difference b/w when you started and now

R; when I started I was very regular and it felt good but now when I smoke I feel like why am I even doing it…but I cannot stop.

I; why do you want to quit

R; it has affected my body..i feel weakness and it has also affected my eyes

I; what do you smoke

R; cigrettes only

I; anything else

R; joint I have tried

I; packet or single

R; earlier I used to buy packet...but now I either take it from my friends or I buy single

I; how many in a day

R; 10-15 …I may start with 4 and if I need again ill buy more

I; food

R; yes I need after food…after whatever I eat I need…after breakfast,lunch and dinner all.

I; do you feel like quitting

R; yes I have tried many times to quit but I could not. Though I have reduced the number.

I; angry

R; many people say why do you do it it is bad…it makes me very angry.. I am not taking money from them to buy it so it should not borther them.

I; guilt

R; no I don’t feel that much guilty… if I smoke I smoke too much if not then it is very less. So no there is no guilt.

I; morning

R; earlier I needed it whenever I woke up

I; night

R; no there is nothing about night as such

I; how many times have you tried quitting

R; once only…that is right now

I; challenges

R; I think that I wont have it in the morning itself…but then I have to do it by the evening or night …no friends don’t influence me…I can easily say no to them but I cannot stop the urge

I; society

R; people my age think of me as a stud and cool when they see me smoking but older people don’t see me nicely

I; your perception

R; it is good

I; tcc

R; yes

I; tcc therapy

R; yes many doctors tell to try nicotex but I haven’t tried it yet if more tccs open then yes it might help people to know its negative effects like cancer

I; any suggestions

R; no I have no idea about it

**Interview**

I; start

P; 5-6 years back…after my 12^th^ standard

I; who influenced you

P; my friends used to do it so I tried it too..i am regular smoker since then…first I had less like 1-2 but now it is more about 5-6

I; only cigrettes

P; yes only cigarette

I; buy and store

P; I buy single-single only and yes sometimes I store them as well

I; any incident

P; places where it is restricted…people tell not to smoke and earlier there was not much strictness in the university camous and I could smoke wherever I felt like but now there is.

I; ever felt likw quitting

P; yes many times.. I don’t smoke at home I only smoke here

I; why cant you quit

P; friends smoke and after watching them I also feel like smoking

I; angry

P; yes I feel irritated when someone tells not to

I; guilt

P; yes many times...

I; emotion

P; whenever I am in a problem I smoke…not when happy

I; timings

P; in the morning itself in the washroom

I; do you wamt to quit

P; yes I am trying to

I; reason

P; I don’t feel like smoking anymore but sometimes it happens…I have enjoyed a lot but now I am stable and I want to stop..it is costing a lot also

I; ever tried

P; yes I have...tried during the covid times..had only 2-3 cigrettes in a span of 9 months.

I; why are you not able to quit then

P; main reason being the friend circle here…everybody smokes so I do it also

I; society

P; here many people smoke so nobody interferes but at home I don’t do it. If it is a no smoking zone so I go somewhere else…nobody says anything

I; timings

P; I smoke more in the evening

I; food

P; after food or with alcohol …after lunch and dinner

I; tcc

P; yes I have heard

I; tcc therapy

P; yes I have heard but never went there..i have heard about chewing gums and I tried…it helped me also but 1-2 times I feel like smoking

I; any suggestions

P; I think cigrettes are very easily available and cheap…ypung people like students can buy very easily..it should be banned

I; what can doctors do

P; doctors can help but I don’t think someone can quit if they themselves dont want to.

**Interview**

I: when u started smoking

A: I started smoking in my11 th class, I started because its fancy and excitement just to see then later I got into ug that’s when I started cigarettes regularly

I: whom

A: one or 2 boys who are like me

I: how many

A: it depends 0n what day and how the day was when its normal day its 4-5, its depends on how my mood is … when its party time 10-20

I: smoking experience

A; I started as I only wanted to try…I feel relaxed when I smoke…I once faced black stains in my sputum

I; access

A; nearby shops

I; packet or single

A; when starting I used to buy single but when I became a regular smoker I bought packets…now I again buy how much are required and not packet … no I don’t store them

I; do you know loose cigrettes are banned

A; yeah I got to know recently but we can still get it

I; control

A; yeah

I; challenges

A; no if I want to I can quit

I; anger

A; in the beginning some used to stop but then they don’t say anything

I; guilt

A; no

I; moring

A; no in the later after of day

I; food

A; yes I smoke after food..it is not mandatory but when I smoke after eating it is nice

After any meal breakfast, lunch and dinner

I; tea or any ither association

A; no

I; quitting

A; yes I tried 5-7 times

I; what is stopping

A; I used to be like it is enough smoking now so I stop for a while

I; improvement

A; I feel more active when I leave…I feel lethargic when I smoke

I; society

A; I know they are judging but I don’t care if they don’t know me…if I know the person then it is different

I; tcc

A; yes I have heard I have myself counselled many pateints

I; tcc therapies you or your friend

A; no

I; any suggestions

A; there should be follow ups and also they should be told about the ill effects properly…more awareness is needed

I; as doctors

A; they can spread awareness and advice them

**Interview :11**

I: when u started smoking

V: 10 days before

I: how

V:I used to sit with my friends then hookah was there , then slowly started

I: where

V: infront of clg

I:how many

V: it was not more or less like normal , 10 minutes

I: age

V:21

I: experience

V: I have started now 10 days before , I doesn’t experienced anything, no impact on me

I: while smoking , how was the experience

V: good

I:access

V: -

I: do u want to quit or not?

V: if friends are sitting doing I will do or I wont

I: have u faced any difficulty while quiting

V: no

I: have u ever angry with ppl who told u to quit the habit

V: when elder person is there , I wont do i will stay away from it

I: have u ever felt guilty

V: no

I: when u smoke

V: evening

I:do u want to quit or not:

V: I will see to quit or continue,

I: why so

V: I don’t think hookah is wrong,its not available 24,7 so I don’t want to get addicted to it

I:society perception

V: it doesn’t matter , what a person think about me ,

I: have u heared of tcc

V: never

I:doctors suggested

V: no

**Interview**

I: when u stTED

R: 2 years ago

With whom

R: with friends

I: where

R: we used to go to parties and we are seeing friends smoking and we felt good about it & I want to try it

I:duration

R: hardly, half hour or 10 friends it depends on friends when I sit with friends it become regular

I: how many

R:12- 15 cigatretes around 1 pack

I:experience

R: sometimes too much cough is coming,sputum is coming out,difficulty in breathing while climbing up and down from stairs

I:access

R: from friends whenever we used to go parties

I:do want to quit

R:sometimes, I thought about it , but this much addidction towards nicotine and I had urge to it lets somke 1 cigarrete and 1 more and I’ll quit later

I:difficulty

R:difficulty in breathing while climbing up and down , I felt relaxed when I sit with 4 friends if the smokes ill also smoke

I:angry

R: they r doing right but at that time we are having fun so we don’t feel good about it

I: guilty

R:yes

I: what time u strat smoking

R:at morning within half hour of waking up

I: do u want to quit

R: hann

I: y

r: im facing facing lot of side effects like inside my body increase in temerapture, lip becaming black, difficulty in breathing while climbing up and down

i: what motivates u to quit

r: sometimes my faces slowly becoming dull and lose its shine

i: what is stopping u to quit

r: im taking more nicotine , IM addicted

i: socity

r:look, closed ones will think about me , for others it doesn’t matter to them otherwise smoking is bad if they are are telling us to stop its for good but we don’t listen to them

i: tcc

r: I never heared about it

i:suggestion

r: try everything but don’t get addicted . there should be a time period then u leave it

i: doctors

r: no

i: suggestion

r: kind of relaxation , when I woke up morning my body feels tired ,no I should to do something then I remember lets smoke cigarretes i will feel ok then when I start to smoke slowly ill get relaxed , nicotine enters my mind then it starts to relax my body but this thinking is wrong we should not do these thinks it has lot of side effects its very harmful for us ,its written on the packet that it will cause cancer but still we do it

**interview**

I: start

R: About 3 years back

I: with whom

R: when I came for my bachelor's in Delhi the with my friends

I: amount

R: Sometimes 2, sometimes 3 or 4

I: Experience

R: Tensions go away

I: Access

R: Friends were smoking so I tried as well

I: control

R: it is already in control but I think of controlling more and quitting

I: Challenges

R: No

I: Guilt

R: yes I feel guilty

When they stop me I feel bad and feel like quitting...If I am smoking somewhere and any friend comes and tells me to stop, I feel bad and then I don't smoke in front of him/her again

I: Morning

R: Yes within 30 minutes to 1 hour of waking up or else in the evening after having food

I: want to quit

R: yes

I: why

R: some health issues I am facing...like breathing difficulty

I: what stops

R: I see other and then I think of smoking

I: what motivates you

R: All the diseases that one gets after or the problems that one may get

I: society

R: I don't smoke in public places and one should not like in hospitals,etc

I: Tcc

R: No

I: Tcc therapy

R: no

I: Doctor

R: No...I want to leave but I can't...I think of leaving it...I won't have it for one day or 2 but then I would want it again

**Interview :14**

I:when did u started smoking

V: 2016

I: with whom, where

V: with friends in kota [rajasthan]

I: how many cigartes in a day

V: 4- 5 cigarettes /day

I: smoking experience

V: it was fun along with drinks, I had sore throat , burning sensation

I: how do u feel

V:I felt dizziness and after drinking water i felt good

I: regular smoking

V:while I was preparing for neet exam whenever I feel sleepy I used to smoke more in night time

I:access

V: shops

I:have you ever felt control

V: whwnwvwer papa sits next to me smell will be coming that time I want to quit , during lockdown , I tried and quit smoking completely then morning I’m like “I want 1 cigarette”

I: angry

V: little angry and im doing my own work why they has to tell something

I: guilty

V:sometimes I feel when I see the older man coughing ,I feel this is not the right age to smoke

I: do u want to quit

V: yes

I: why

v:while doing physical excersise , I faced problem in breathing

i: why u rnot able to quit

v: friends are smoking and they offered me I can’t resist

i:society

v:doesn’t matter

i:know about tcc

v: before I dint know when I came to nims I get to know

i: what u know

v: doctors only know

i: any therapy u undergone in tcc

v: no

i:any doctors have adviced to quit smoking

v: no

i: anything

v: upcoming generation should not smoke its injurious

interview :15 raghul goswani

i: when u started smoking

r: I started smiking in 2018 when I was in coaching

i:where

r: with friends while partying in sikkar [place]

i:what age when u stared smoking

r: 17- 18

i: how man cigar in a day

r: in starting 1o r 2 then addidcted to it ,1 packet /day

i: experience

r:while partying it was fun

i: access

r: shop

i: have u ever control smoking

r: im addicted to it so im not thinking about quitting

i: angr e

r: I smoke with my money I don’t care about them

i: guilty

r: in starting , I felt guilty then I get used to it , I cant leave this and life is going

i: whattime

r: in morning its compulsory afterthat ……..

I: do u want to quit

R:hannnn I want to quit but never tried

I: why u want to quit

R: long time back . cough is coming sometimes in winter I had cough

I: which Is stopingu to quit

R:because of addidction im not able to quit

I: society

R: Im living in the society where ppl wont smoke and they wont allow me to smoke, im not able to smoke in my home so I want to quit

I: know about tcc

R: hannn when I came to know nims denta college

I: wht

R: in tcc they help to qiit smokimg within 30 days

I: suggestion

r: I don’t know

i:has any doctor suggested u

r: -

i: anything

r: initially, I thought it was fun and it has lot of benefits, sutta dhuvan dhuvan [ while smoking ] inside me , I felt good . and somewhere else slowly slowly , Im addicted to it and I realized its bad for society

while smoking ,I don’t faced any difficulty afterthat I felt difficulty in running ,gym activities, while doing heavy work I faced breathing problem

**INTERVIEW :**

I: start

15: in 2018-19, when I was having my coaching...I tried it with my friends in a party in Sikar

I: age then

15: 17 or 18

I: frequency

15: in starting I used to have 1 or 2 but then I developed this habit and then I started having the entire packet (10 cigarettes) in one day

I: Experience

15: I tried it for the first time with my friends at a party for fun...it felt good

I: access

15: nearby stalls or shops

I: control

15: It is a habit now so I have not thought of quitting or no

I: angry

15: No i care what other say...I am having it with my own money

I: guilty

15: No... In the beginning I used to feel guilty but not now...it is habit and I cannot live without it now

I: time

15: in the morning I need it...1 cigarette is compulsory...then during the day I have it with tea..

I: want to leave

15: I can leave but I have never thought of quitting

I: why leave

15: it's been a long time since I've been smoking...so I cough a lot ...I have difficulty in breathing also...in winters I sometimes experience mucus also

I: what stops

15: it is a habit and I am addicted now...so I can't leave

I: factors

15: there are many people who don't smoke...also my family doesn't allow it

I: society

15: Yes that's why family doesn't allow and I cannot have it when I am at home so I want to quit

I: Tcc

15: Yes when I came in NIMS University I got to know about it that they can make you quit smoking in 30 days

I: Tcc therapy

15: No

I: Suggestion

15: I am here for the first time...I don't know

I: doctors

15: Yes my relatives and friends also...told me to quit...they also said that it wouldn't be nice if your family got to know about it...No i haven't heard it from a doctor to quit

I: anything to add

15: In the beginning we only see good...smoke and all...it feels like a good way to get lost in yourself but ones it becomes a habit...it is bad...I enjoy smoking but afterwards I have problems like I cannot run or go to gym...any tedious work I cannot do.

**interview**

i: when u started

d: in 2022

i: where

d: with friends during partying

i: how many cigarettes

d: 1 cigarretes/day

i:experience

d: as of now I don’t feel anything good , when i saw my frnds smoking i also starts

i:how did u feel

d:in starting I got headache

i:accessd

d:I don’t buy on my own I get it from my friends

i: control

d: I don’t smoke much so Its not needed

i:difficulty

d: I never faced any difficulty even if I don’t smoke 2-3 days

i: angry

d:not like that

i: do u want to quit

d: yes , when I sit with my friends gain I started with cig

i:what timing

d: night time when I go for tea along with tea

i: why u r not to quit

d: I tried and I didn’t smoke for some days

i:what is stopping u to quit

d:theres nothing like that when I sit with my frnds ill do

i:what factor motivates u to quit

d: when I see the other ppl around me, depressed ppl , health issues like asthama ,cough, society also telling bad habits and doesn’t have any benefit but Iike to smoke

i: society

d:it matters we are in good profession its bad for me and for the society too we are setting abd ex for society

i:tcc

d: I don’t have any idea

i: what u

d: its for quitting bad habits like ghukta

i: any therapy u underwent

d:no

i: suggestion

d: the should suggest to quit slowly slowly by some gums not in sudden

i: doctors

d: no , I know its harmful

i:anything

d: I want to tell something to everyone there’s no benefit, health will be spoiled, harmful environment to environment , a person whoever is in front of me who doesn’t smoke also gets affected .

**interview**

I: start

S: In first standard, with my friend at his house...his father's half smoked cigrette was lying and we burnt it to try.

I: Age

S: 24 years

I: Frequency

S: Right now it is 2-3 cigrettes per day

I: Experience

S: Sometimes when I feel that I cannot handle the stress...it is destressing

I: access

S: Asked my friends who smoked to get it for me

I: Control

S: Not right now

I: Problem

S: No...but I know that I should not do it

I: Angry

S: I don't get angry if someone criticize me...I get irritated when someone tells me what to do and what not. If someone properly advises me then I will consider it

I: Guilty

S: no...not now

I: timings

S: in the day...after college or in between college

I: want to leave

S: Not right now

I: why

S: The stress that makes me smoke isn't over yet

I: What stops you

S: Stress and sometimes anxiety or if I don't have anything else to do

I: What type of anxiety

S: Now I don't have a reason...I get anxiety pangs and I smoke

I: what motivates you

S: Good skin... People who don't smoke have good skin..they look good also...It may also be genetics but people say that smoking makes your skin bad

I: Why can't quit

S: Because I haven't decided yet...if I decide I can quit...Now I feel it is fine..I know eventually I will have to leave but I feel the need of it sometimes.

I: society

S: Not only about smoking... I never think what people think about me. But yes if I cross by an elderly person while smoking who is about the of my parents, I'll hide my cigrette...this is what I have learnt from elders...out of respect... whether I know the person or not ... Like I do not smoke in front of my parents so there is no point..

I: Does it matter to you what do they think

S: No their opinion does not matter to me...It's just my principles that I hide it in front of elderly

I: Tcc

S: Yes I know but I haven't went there ...I know they counsel their to quit smoking...that's all I know

I: Tcc therapy

S: No

I: Tcc Suggestions

S: I never went there in the first place

I: what can doctors do

S: I haven't seeked any help regarding quitting

I: anything you want to add

S: if we have to solve this problem then we must go to the foundation of it...taxes and all is a temporary solution...when people feel the need of it... they will get it from anywhere.

Also, I advise people not to smoke, if they haven't started yet.

**interview**

i:when you started smoking ? at what age ?

p: at 23 years

i: what you have smoked how its stareted ?

p: cigarettes

i: any reason why you started ?

p: started just like that ….. mostly due to emotional downfall

i:when you started frst ?

p: it was with friends.

I: friends with whom you have smoked also a smoker ?

P: yes

I: how much cigareetes you started smoking ?

P... Initially, It was less. I started smoking in pune was planing to go goa, then tried it felt good. Initially I was having 2- 3 cigarettes.

I: what you felt good ?

P: I like the nicotine rush …I consumed the frst cigareete of the day I feel the head rush and that was amazing

I: how many cigareetes did u consume in ad day ?

P: 10

I: any smoking expeirenece you wanna sharev ?

P: my shirt gets spoiled I gets burned

I:how you get access to cigarettes ?

P:shops ,tapris

I: have you ever felt to control your smoking ?

P:many times … I felt that but cant do it …

I: what kind of difficulties u hav effaced ?

P:not able to control I feel very annoyed

I: how many times you have tired quitting ?

P: may be 1 time during covid 19

I: have you get annoyed if someone crticies your smoking habit ?

P: no I don’t care

I:have you smoke after you wake from bed ?

P: it depends on the avilabiloty .. if im getting cigerattes for 24 hours I will smoke for 24 hours ,….if I wont get I wont smoke as simple as that ..

I: what time of the day you smoke more ?

P: 4 ‘o clock in the evening with chaii.

I: why you are not able to quit ?

P: nicotine addiction .. bro

I: have you ever tried to quit smoking ?

P: I tried for 15 days soryy I think 10 days but later I started

I: why you started again

P: because my friend was smoking and he has cigarettes so I also started

I:how society see u when you smoke

P: ***** (bad abuse words )

I: have you heared about TCC theraies?

P: I heaqred about nicotine patch

I: any suggestions to promoite TCC ?

P: im not getting motivation from tcc , people are naïve and they don’t anything about it….

**interview**

I: start

18: When I was 13 with my friend at his house

I: Duration and Frequency

18: 3-4 in a day

I: Experience

18: While exercising or running I feel difficulty in breathing

I: access

18: My friend got it from a shop

I: control

18: I feel like this daily...I try to quit but I can't

I: challenges

18: Nothing I just feel like smoking

I: Angry

18: Yes...a little bit...when my family or friends stop me...why are you worried whatever it is, it is with me only

I: guily

18: yes I feel sometimes that I should not do it

I: time

18: After having food... mostly at night

I: what to quit

18: yes...

I: why

18: I am having breathing difficulty...It also smells bad...my family also doesn't like it

I: what stops

18: I just can't stay without it...can't let go of it

I: what motivates

18: family and friends

I: society

18: society sees me as a cringe person or a rouge. They may think of me like someone who doesn't have manners or has nothing else to do.

I: Does it affect you

18: No... people speak anything

I: Tcc

18: no

I: Tcc therapy

18: no

I: doctor

18: whenever I have a problem and I go...they all say no...

I: suggestion

18: One should not do it...it is a bad habit...if you start it is difficult to leave also. Listen to your family.

**Interview**

I: start

19: I was in my internship and with one of friends I said ok I'll try but not that much...after I came here like...1 year back...I started smoking.

I: what age when you started

19: 24-25

I: frequency

19: earlier I used to share...now also I have with my friends

I: duration

19: 10 mins maximum

I: Experience

19: One bad experience is there, Once I was at my friend's place and it was winter time...we were smoking and I was wearing a puffer jacket and I started sweating so I went outside, in the balcony...then I fell unconscious...

I: Control

19: Yes

I: difficulty

19: My friends are always there surrounding me...and if they are smoking, I'll also take and smoke

I: what makes you think of quitting

19: I was bored...I was like ok smoking is enough now

I: angry

19: Just because I feel like they should mind their own business

I: guilty

19: yes I feel guilty that I should stop smoking

I: timings

19: Morning, when I get up...that time I smoke..if I don't have any I won't smoke...if there is cigarette in my room then i'll smoke...then in the whole day there are options

I: Now you want to quit

19: yes because it is bad

I: why?

19: because I am loosing weight...loss of appetite is there... weakness is there

I: what stops you

19: (laughs) Maybe it's me...I am not quitting it

I: society

19: yes they see us and they judge us so it is obviously is not good

I: Tcc

19: yes...you can counsel there... people who are addicted to tobacco

I: Tcc therapy you or your friends

19: No

I: improvement suggestions

19: I think people will not quit until and unless they want to quit... how much ever you counsel and tell them they won't stop ..until and unless they themselves want to stop it.

I: has any doctor who knew about it, helped you?

19: no

**Interview**

I: start

20: I started when it was my last day of internship...we were partying and I smoked once with my friends...that was the starting...my age was 24 that time

I: frequency

20: daily 5 to 6

I: duration

20: from morning till evening it's like 1 or 2...after evening it becomes more

I: experience

20: when I started that time I had only one then I left it...now since past 1 year I started smoking...so in this time...I usually smoke with my friends...it is a good experience.

I: access

20: I go to shop and buy it

I: control

20: yeah sometimes...when I am stressed out I crave for cigarettes...

I: difficulty

20: difficulty, I think... Might be...I get pissed off...at my work or if there is so much anxiety or else anything if I crave for cigarette at that moment it creates issues but nothing like that...sometimes..that is very unusual

I: angry

20: yeah I think like it is my business...don't interfere in my business...I will advise them to mind their own business

I: guilty

20: no I don't feel guilty Because I won't smoke that much that I feel guilty about it

I: time

20: morning I'll start definitely but till afternoon it is very less like 2-3.

I: do you want to quit

20: yes I want to quit because I feel like money is going like water (snaps) ...for economic purpose only I want to stop...rest nothing

I: what stops you

20: because people around me smoke I can't think of quitting that only

I: society

20: yes sometimes they judge and that looks odd to me but it's ok...if now I can't get rid of it...after sometime I'll definitely stop because I know I have that much self confidence that I'll get rid of it

I: Tcc

20: yeah I know... addicted people come in Tcc and doctors are there who advise and counsel...and lead people to non smoking area...that's good actually

I: improvement suggestions

20: we should make more awareness about it... because these days because of smoking only... carcinomas and all is getting increased...so people should be aware and those who are severely addicted they should atleast quit some portion of it because it is causing health hazard I feel if there are camps or any social gatherings like if we influence people like that only...it can help

I: Tcc therapy you or friends

20: no

I: any doctor

20: no

I: any add on

20: actually I want to say that it is obviously not good for health...but sometimes it relieves stress so there is a positive effect and there is a negative effect...I feel like so I will try to quit (smiles)
