# Supplementary material for: Beyond the puff: qualitative insights into smoking behaviours and societal perceptions among university students in India
Source: BMJ Open. 2025 Jun 24;15(6):e101172. doi: 10.1136/bmjopen-2025-101172 (PMC12198830; doi:10.1136/bmjopen-2025-101172)
Supplement: Supplementary file 3 [file bmjopen-2025-101172.reviewer_comments.pdf]

PEER REVIEW HISTORY

BMJ Open publishes all reviews undertaken for accepted manuscripts. Reviewers are asked to complete a checklist review form and are provided with free text boxes to elaborate on their assessment. These free text comments are reproduced below.

ARTICLE DETAILS

Title (Provisional)

Beyond the Puff: Qualitative Insights into Smoking Behaviours and Societal Perceptions among University Students in India

Authors

T, Seemadevi; S, Naganandini; chhabra, Kumar Gaurav; Chaudhary, Pankaj; Singh, Avishek; Dangi, Shweta

---

VERSION 1 - REVIEW

**Reviewer**	**1**
**Name**	**Surarong Chinwong**
**Affiliation**	
**Date**	**11-Nov-2024**
**COI**	**No competing interest**

Comments to the Author
Thank you for the opportunity to review this manuscript. There are several points that require clarification or further consideration:

1. The study's objective was to understand adult smoking behavior and the influence of societal perceptions on it. While the qualitative approach using in-depth interviews is appropriate, there is a notable lack of diversity in the participants' fields of study. With 14 out of 20 participants from healthcare professions (pharmacy and dental), 5 from engineering, and only 1 from law, the data may not be sufficiently diverse to comprehensively explain smokers' behavior and perceptions. Could the authors elaborate on how they ensured data saturation and why they did not include participants from a broader range of academic disciplines?

2. The authors state that the study employed a hybrid approach, combining inductive and deductive methods for analysis. However, the implementation of this approach is not clearly delineated. A more concrete demonstration of this methodological approach would strengthen the paper's methodological rigor.

3. The study's original contributions and practical applications require further elucidation. The authors should more explicitly articulate how their findings advance current knowledge in the field and how these results can be translated into practical interventions.

4. Given the limited sample size of 20 students from a narrow range of professional fields, the generalizability of this study is questionable. While the authors suggest that their findings can inform the development of targeted smoking cessation programs for college students, this claim may overstate the implications of their results. A more detailed discussion of how these findings might be cautiously applied or used to inform future, more comprehensive studies would be beneficial.

**Reviewer**	**2**
**Name**	**Smith, Marissa**
**Affiliation**	

**Date**	**11-Nov-2024**
**COI**	**No competing interest**

Comments to the Author
Major comments
1. The methods lack detail on several points including sampling, recording of demographic data and coding. The interviews last 10-15 minutes and I am interested to have more details on this. Were longer interviews requested but the participants only spoke this amount of time? I have included more comments in the minor comments sections.
2. The results requires major revision. Theme 1 is listed within a table and there is not discussion about the quotes. In Theme 2, numerous points are made but not quotes are provided to illustrate these points. Typically, a point would be made with a quotation to support this. Theme 3 is missing. Theme 4 is set out in this way, I recommend revising the other themes to follow a similar structure.
3. Quote attributes are unhelpful. "20s/male" Is the 20s referring to age? Please amend these to be more meaningful e.g. the participant age.

Minor comments
Abstract
1. Define NIMS.
2. Please include when the interviews were conducted.
3. Please include age range of participants.
4. I would recommend changing "snowball and purposive sampling" to be the other way round. I would assume you used purposive sampling followed by snowball.

Introduction
1. "According to the World Health Organization (WHO), tobacco smoking is responsible for 4.9 million deaths each year."- Include citation. Is this a recent figure? In the next sentence, the authors state the figure is expected to double by 2030. It is unclear if the figure is expected to double from now (2024) to 2030. More detail is needed.
2. "According to the Global Adult Tobacco Survey 2016-2017, 42.4% of men, 14.2% of women, and 28.6% of all adults currently either smoke tobacco or use smokeless tobacco." These stats are from 7/8 years ago. Please include more update stats.
3. "Young people are the most valuable target for tobacco companies because, due to the addictive nature of tobacco products, attracting them to start smoking ensures many years of continued consumption, making it an exceptionally effective long-term investment." – This is an interesting statement; however, the authors then go on to discuss stats relating to young adults and adults which is then the focus of the paper. This statement is somewhat out of place. If the authors wish to include this then more context is needed as to its inclusion.
4. The authors use the terms young people, adolescents and adults in this section. It is confusing to the reader as this can relate to very different groups, please use consistent terms.
5. "The results of this study can serve as a foundational source for developing health programs focused on these areas, helping to eradicate smoking in adults."- I recommend removing this from the introduction and ending this section with the aim.

Methods
1. Change clearance to approval.
2. Define NIMS.
3. How were participants selected from outpatients? Please include more details on the sampling of participants.
4. Check capitalisation at start of sentences.
5. Why was an oral cavity check required? More detail on this is required. Who conducted the check and when?
6. How was demographic information collected, a questionnaire? What demographic data was collected?
7. "The face-to-face interviews included questions about participants' age, gender, year of study, and their level of tobacco dependence, assessed using the Fagerström test for Nicotine Dependence level [FTND]scale for smoking."- Were some of these details not recorded when collecting demographic data
8. Please include a copy of the topic guide as an appendix.
9. Define CAGE.
10. 10-15 mins is very short for an interview. Why was this time period selected?
11. Did any double coding take place? Were there any issues relating to the coding that required to be resolved?
12. Were participants given any incentive?

Results
1. "A total of 20 participants were interviewed for the study, 15 male and 5 female."- Include percentages.
2. Was the professional course stated by participants during demographic data collection? If so, please include this in the methods section about this.
3. Please include details on the split of ages of participants.
4. The authors describe data saturation and it is interesting that only five females were included in the study. Did the authors consider extending recruitment to include more females?
5. Please include n=x when reporting percentages.
6. I don't think the use of gender is correct here when only male and female are used. Did the authors ask about biological sex or gender? If it was gender I would expect more than two categories.
7. How was dependence determined?
8. I recommend unpacking some of the themes in Table 3 rather than just listing them. What are some of the keys points from these?
9. Please include quotations to back up the points being made in Theme 2.
10. Theme 3 appears to be missing.
11. The organisation of Theme 4 is how the other themes should be structured.
12. "Gender and smoking" – I believe the authors are referring to sex not gender.

Discussion
1. "India is the leading producer of tobacco, and a significant portion of its young adult population is addicted to this habit, largely due to tobacco companies targeting the adult college students [12]" – This is would more suited to the introduction as it is not clear the study is conducted in India to the methods. Furthermore, the title of the paper could be amended to indicate this focuses on adults in India.
2. "Some qualitative studies also showed the smoking behaviour in adults they highlighted that stress is an important factor attributed to smoking behaviour and results consistent with our study findings [15]."- This sentence doesn't make sense.
3. Please include more discussion on the context of the findings with wider research.
4. The discussion is lacking crucial discussion points such as strengths and limitations, implications for policy and practice and future research recommendations. Please revise the discussion to include these.
5. It would be beneficial to include some discussion on reflection on the role of the researcher.
6.

Conclusion
1. I recommend revising the conclusion to be stronger. It would be beneficial to include reference to policy implications.

VERSION 1 - AUTHOR RESPONSE

I have addressed all the revisions suggested by both the reviewer and editor.

VERSION 2 - REVIEW

**Reviewer**	**1**
**Name**	**Surarong Chinwong**
**Affiliation**	
**Date**	**7-Jan-2025**
**COI**	**No competing interest**

Comments to the Author
Thank you for the authors' comprehensive response. Several points warrant further consideration:
1. Would the authors kindly elucidate their methodological approach by providing a specific example or detailed protocol of their inductive theme generation process, particularly demonstrating how these findings complemented the deductive analysis of the CAGE behavior scale?
2. While the clarification regarding recruitment procedures and acknowledgment of sampling limitations is

appreciated, there appears to be a notable methodological concern regarding participant diversity. The demographic distribution reveals that 70% of participants (14 out of 20) originated from healthcare-related disciplines (pharmacy and dental), whereas engineering and law contributed only five and one participants, respectively. This concentration within healthcare-related fields potentially introduces sampling bias and may inadequately capture the broader spectrum of societal perceptions across diverse academic disciplines. Although data saturation was achieved, the absence of participants from disciplines such as the humanities, business, or social sciences raises questions about the generalizability of findings to the broader collegiate population. Could the authors elaborate on their data saturation criteria and address the rationale for not incorporating participants from a more diverse range of academic disciplines?
3. The literature review would benefit from a more comprehensive examination of extant research regarding determinants of smoking behavior among university students and related societal perceptions. Furthermore, a detailed comparative analysis between the present study's findings and those of previously cited studies would significantly enhance the discussion section.

**Reviewer**	**2**
**Name**	**Smith, Marissa**
**Affiliation**	
**Date**	**7-Jan-2025**
**COI**	**No competing interest**

Comments to the Author
Thanks for including all revisions.

VERSION 2 - AUTHOR RESPONSE

The following changes and clarifications have been made
